# Multi-scenario simulation of carbon stock and landscape ecological risk changes in Jinpu new area and analysis of spatial conflict relationships

**DOI:** 10.1038/s41598-025-03148-8

**Published:** 2025-07-01

**Authors:** Jin Peng Wang, Li Wang, Peng Du

**Affiliations:** Liaoning Normal University, Dalian, China

**Keywords:** Carbon stocks, Landscape ecological risk, PLUS model, Spatial conflict, INVEST model, Land use change, Ecological modelling, Ecological networks, Ecosystem ecology, Ecosystem services, Ecology

## Abstract

Sustainability of carbon sink capacity is an essential player in the health of the city, and landscape ecological risk can reveal present status of land management. This paper analyzes the distribution of spatial conflict areas between high carbon stock and landscape ecological risk under the current scenarios and future three different development scenarios in 2025 and 2027. The results showed that: (1) from 2019 to 2023, the higher carbon stock areas and the ecological higher-risk areas generally expand; (2) from 2025 to 2027, under different development scenarios, higher carbon stock and higher-risk areas showed different changes, according to the distribution of spatial conflicts between the two, the significant common zones of conflict were distributed in the central, southern, and southeastern parts of the study area, while in the northern and eastern parts of the study area, the spatial conflict distribution of the natural development and cropland protection scenarios is significantly lower than the urban development scenarios, so future land use development patterns can be optimized by combining the respective characteristics of natural development and cropland protection scenarios. Therefore, this study can provide a basis for the future economic development of study area and the green and healthy development of Jinpu New Area.

The future sustainable green development of cities is heavily reliant on drawing a red line between ecological protection zones and other land categories, rationalizing each category’s geographical distribution, altering land use planning, and maintaining ecosystem balance^1,2^. Restructuring the hierarchy of spatial planning and improving China’s spatial governance both need ecological spatial demarcation^3^. Because the carbon stock acts as an important connection between socioeconomic and ecosystems^4,5^, altering land use to decrease carbon emissions remains a national goal in the disciplines of research and technology^6^. The national carbon peak action program for 2023 comprises specific green, low-carbon carbon reduction initiatives. Given the high speed of urbanization and the consequent tensions, we must also pay attention to the expansion of the economy while maintaining the capacity of the carbon sinks, which is prominent to accomplish land-use intensification and lasting development^7^. Rational land use patterns can benefit the environment and the economy, whereas unreasonable land planning can violate the laws of the natural world^8–10^.

Landscape ecological risk is the possible negative effects of the interplay of ecological processes and landscape patterns under the impact of anthropogenic or natural factors^11^, and is an important method of coupling human-land relations from the landscape scale^12,13^. The indicator, which has been used by numerous academics and is a key indicator for assessing ecological quality^14–16^, is based on changes in landscape pattern changes and is used in conjunction with the ecological environment’s carbon stock density to measure the health of the ecosystem^17–19^. The ecological risk of urban landscapes reflects, to some extent, the spatial variability of the urban environment^14^. Terrestrial ecosystem serves as a miraculous role in maintaining the global carbon cycle^20^ and land use changes have altered the framework and cover of terrestrial ecosystem^21^. Although many scholars have analyzed and calculated the two ecological indicators according to the land use situation, most of the existing studies only analyze the carbon stock and landscape ecological risk indicators^22–24^, which is still insufficient, such as the interaction between the two in the spatial pattern of ecological security and the potential land use irrationality reflected by the interaction is still less explored.

Spatial conflict denotes that the incongruity of distribution in space of research targets, meaning that the distribution of one element should not or should appear less in the spatial location of another element. Landscape ecological risk and carbon stock, are directly affected by land use changes. The spatial distribution of land use types within the region is judged to be in conflict through the relationship between the two indicators in terms of their influence on the respective indicators on different land use types, thus reflecting the spatial conflict relationship between the two indicators. As the main source of maintaining carbon density, the maintenance of carbon sink capacity is intimately connected to the maintenance of city forests, and according to the study area geographical differences, in study, the higher ecological risk is set in the cultivated land (including wasteland and unutilized land) and grassland are the major cause. Higher or lower ecological risk is correlated to the management of cropland and grassland, and the high carbon stock density area has a direct relationship with the distribution of forests, so the expansion of the ecological high risk area will inevitably affect the reduction of forests, thus affecting the function of the urban carbon sink service, so in this paper we define the clustering area of high and higher carbon stock area and high and higher ecological risk area as the spatial conflict area. Conflict areas should maintain a basic carbon sink capacity to avoid wastefulness in the process of disorderly development and utilization of land, the continuous expansion of ecological high-risk zones, the weakening of ecosystem carbon sink functions, and the expanding areas of conflict, which exacerbate the ecological risk of imbalance within urban ecosystems. Based on the principle of spatial conflict generation.

This paper utilizes the spatial conflict distribution relationship on the interaction to reflect and explore a reasonable land use development mode, to maintain green development while promoting economic growth, to maintain urban carbon stock while reducing or moderating the increase of ecological risk, taking Jinpu New District as the study area, Jinpu New District, as one of the national new districts in China, has a huge development potential in the future, and the land planning becomes one of the primary issues to be Land planning has become one of the primary issues to be considered in the development process. It takes the end of “the China’s 14th Five-Year Project” and the starting of “the 15th Five-Year Project” period as the time points to research the land-use change may impact on carbon stock and landscape ecological risk. Jinpu New Area has a high land utilization rate, little arable land per capita, and the human-land conflict is becoming more and more prominent, which is in the status quo of too many people, too little land^25^, and significant land conflict. Based on the two indicators, we predict the conflict situation under different land use scenarios in the future according to the current scenario, so as to provide a reference basis for the future development of the city to adopt a suitable land use development mode, reduce land conflicts, build ecological barriers^26^, and maintain ecological land balance^27,28^.

## Introduction to the study area and data sources

### Introduction to the study area

**Fig. 1:**
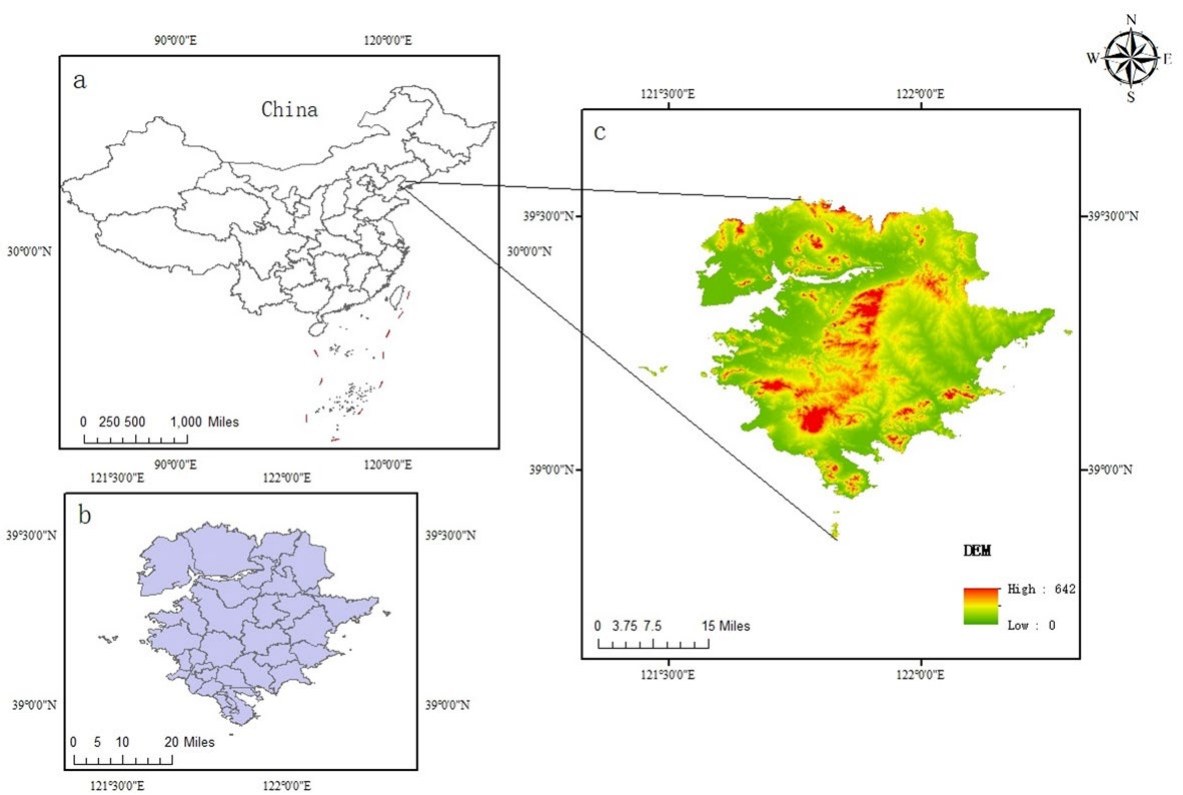
Location of the study area.

Jinpu New District (Fig. 1), belonging to Dalian City, Liaoning Province, covering all the administrative area of Jinzhou District and part of Pulandian District, Dalian City, was established in Northeast China’s first state-level new region and the country’s tenth overall., the total area is about 2299 square kilometers. And it is the largest new area in terms of land area among the 19 state-level new areas, with a resident population of 1.61 million. Dalian Jinpu New Area is located in the geographic center of Northeast Asia, backed by the vast Northeast China, bordering the vast Yellow and Bohai Seas, with a suitable climate, rich ecological diversity, superior geographic location, convenient sea and land transportation, and great potential for economic development, it is the Northeast China’s air and sea gateway to the world, and an important hub for economic and trade exchanges and opening up of cooperation with Northeast Asian countries^25^.

(Figure 1 shows an overview map of the study area, (1) a represents a map of China, which is located in Dalian City, Liaoning Province, China, (2) b represents the latitude and longitude location of the study area, (3) c represents the DEM elevation profile of the study area).

### Data sources

The 2019, 2021, 2023 land use data used in this paper comes from the Google Earth Engine (https://earthengine.google.com/) 10 m high-precision Sentinel-2 MSI Level-2A data, based on which the datasets are interpreted and the land is classified into five categories: construction land, water, forest land, grassland and cropland. DEM data spatial resolution is 90 m from Geospatial Data Cloud (https://www.gscloud.cn/); Population density data from the World Pop (https://hub.worldpop.org/); Road network data from (https://www.openstreetmap.org/) GDP data from (https://stats.dl.gov.cn/col/col3811/) Administrative division data from (https://docs.gmt-china.org/latest/dataset/gadm/index.html).

**Fig. 2:**
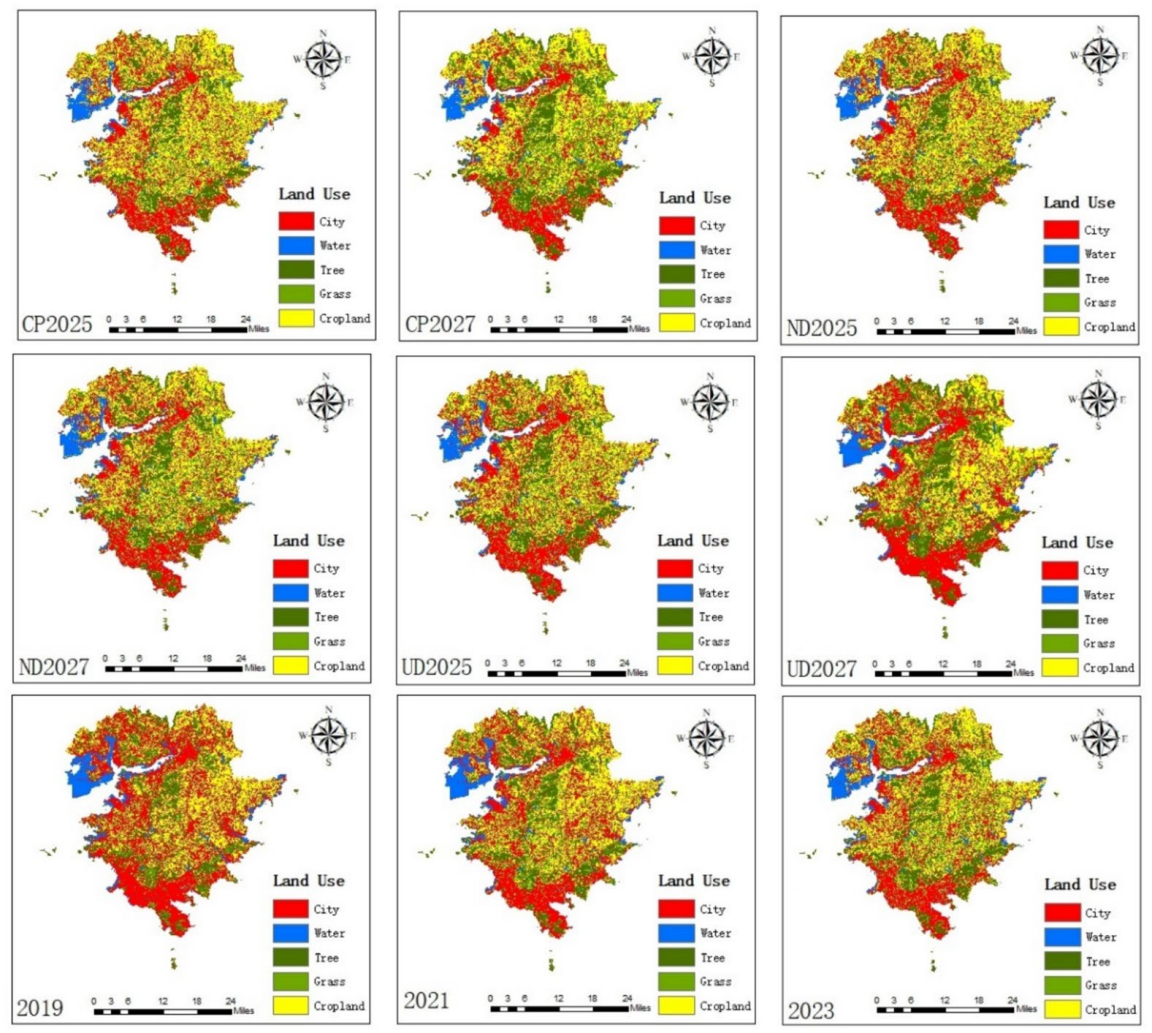
Spatial distribution of land use in different years. (Figures 2 and 3 show land use results with changes in area by type under different development scenarios for the current years 2019, 2021, 2023 and the projected simulations for the years 2025 and 2027. (1) From 2019 to 2023, there is an overall decrease in the area of city and water, a flat change in grassland, and an overall increase in the area of forested land and cropland, (2) from 2025 to 2027, there is little change in the area of the overall land categories in the natural development scenario, a significant increase in the area of city, a smaller increase in forested land, and a decrease in the area of the other land categories in the urban development scenario, an increase in the area of cropland in the cropland preservation scenario, an increase in the area of cropland in the No significant change in city and a significant decrease in other land categories).

**Fig. 3:**
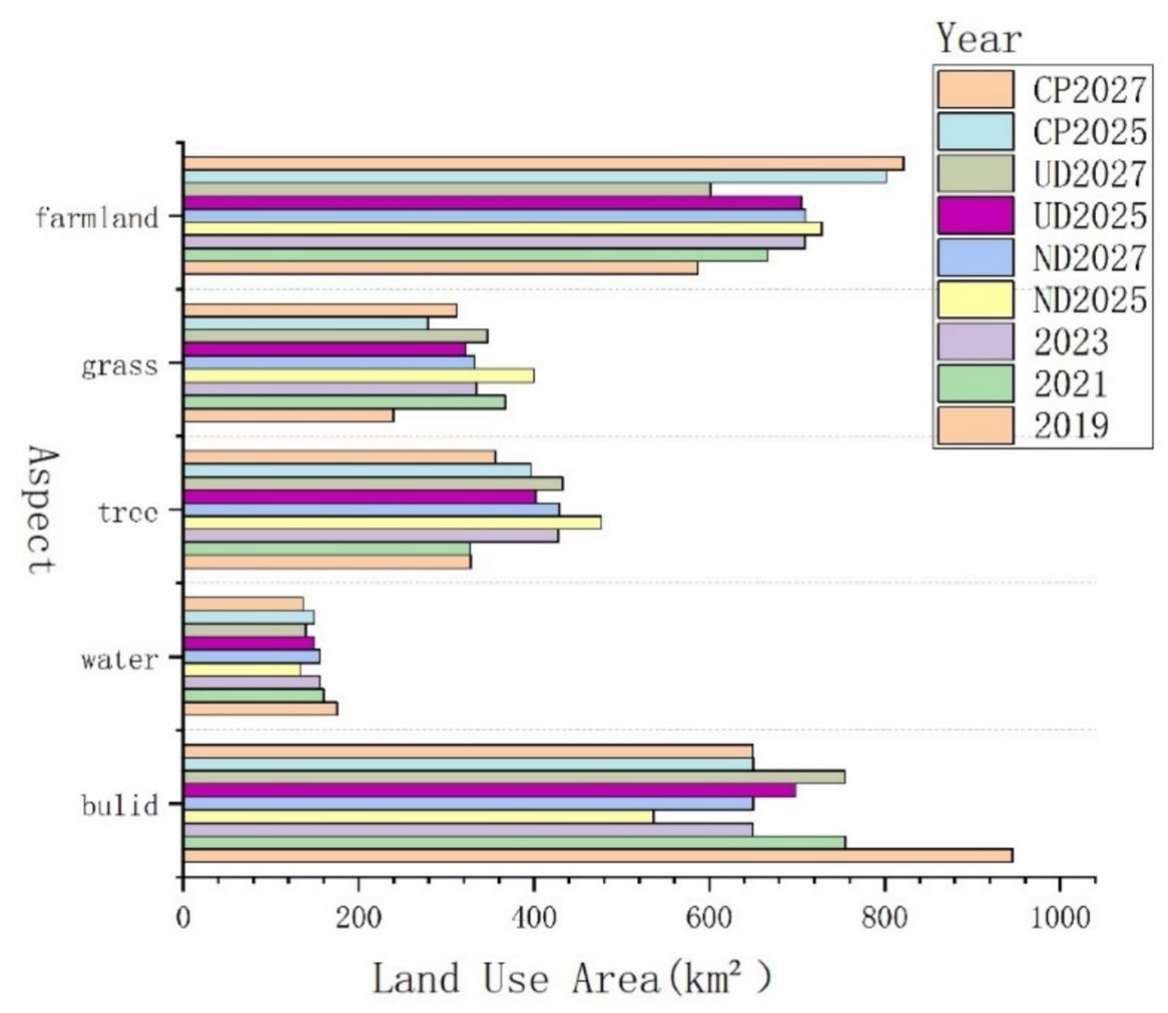
Changes of land use area in different years.

**Fig. 4:**
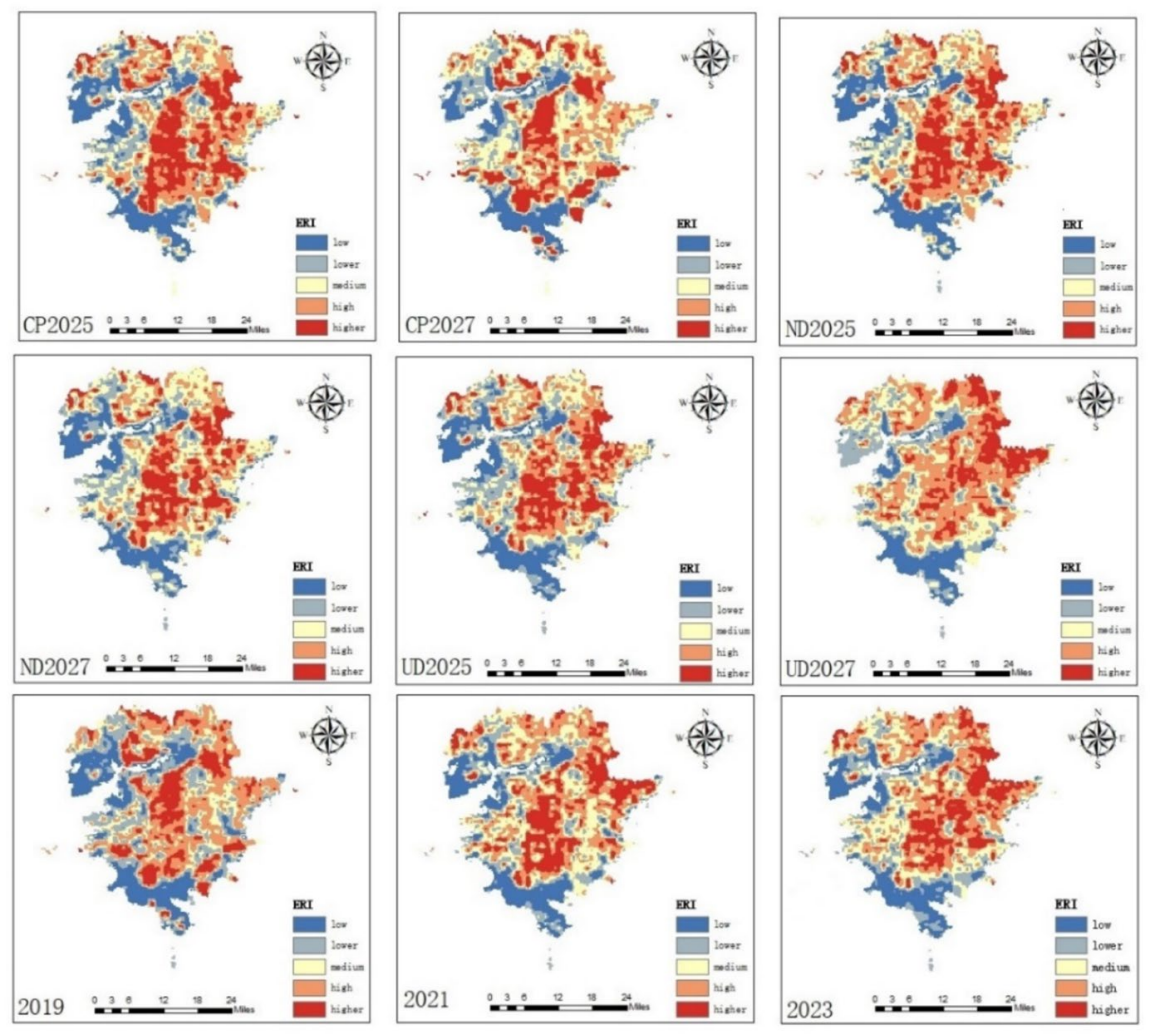
Spatial distribution of ERI in different years. (Figures 4 and 5 shows the changes in ERI under different development scenarios in 2019, 2021, 2023 and 2025 and 2027 obtained from the forecast simulation, (1) the area of high-risk zones increases significantly from 2019 to 2023, concentrated in the central and eastern regions. (2) Under different development scenarios, the ecological higher-risk areas generally expand in cropland protection scenarios and decrease in natural development and urban development scenario, the increase in the high-risk areas of the cropland protection scenario is more pronounced than the other two. Differences in the distribution of high risk across scenarios are evident in the central, northern and northeastern regions).

**Fig. 5:**
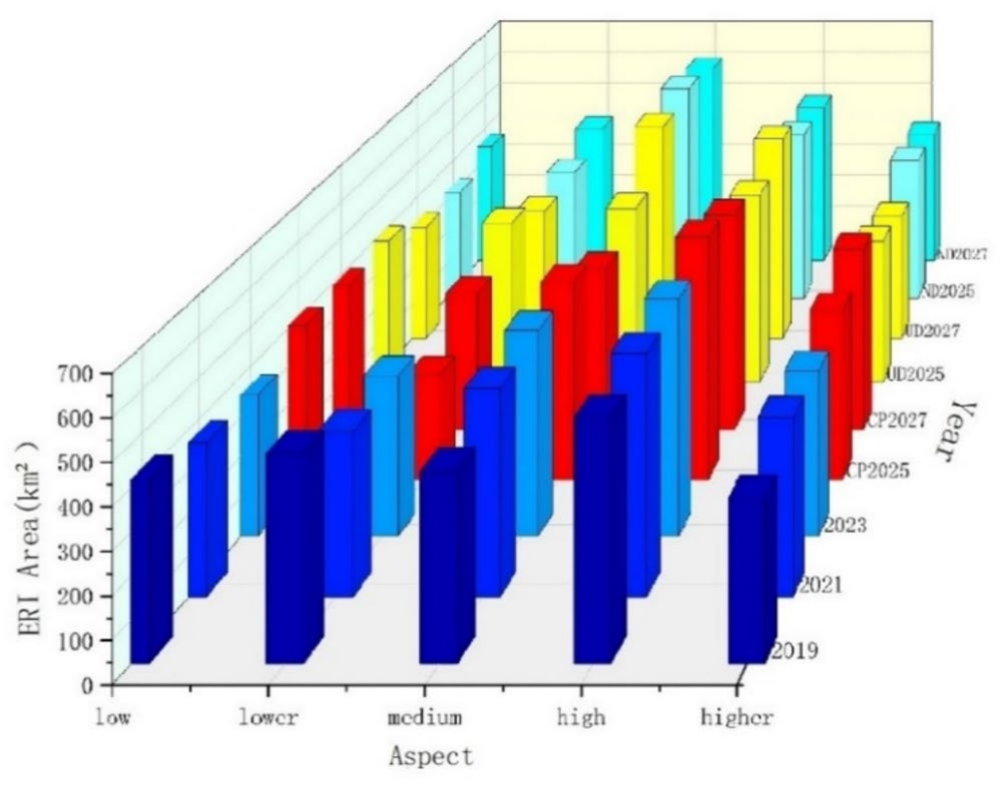
Changes of ERI area for different years.

**Fig. 6:**
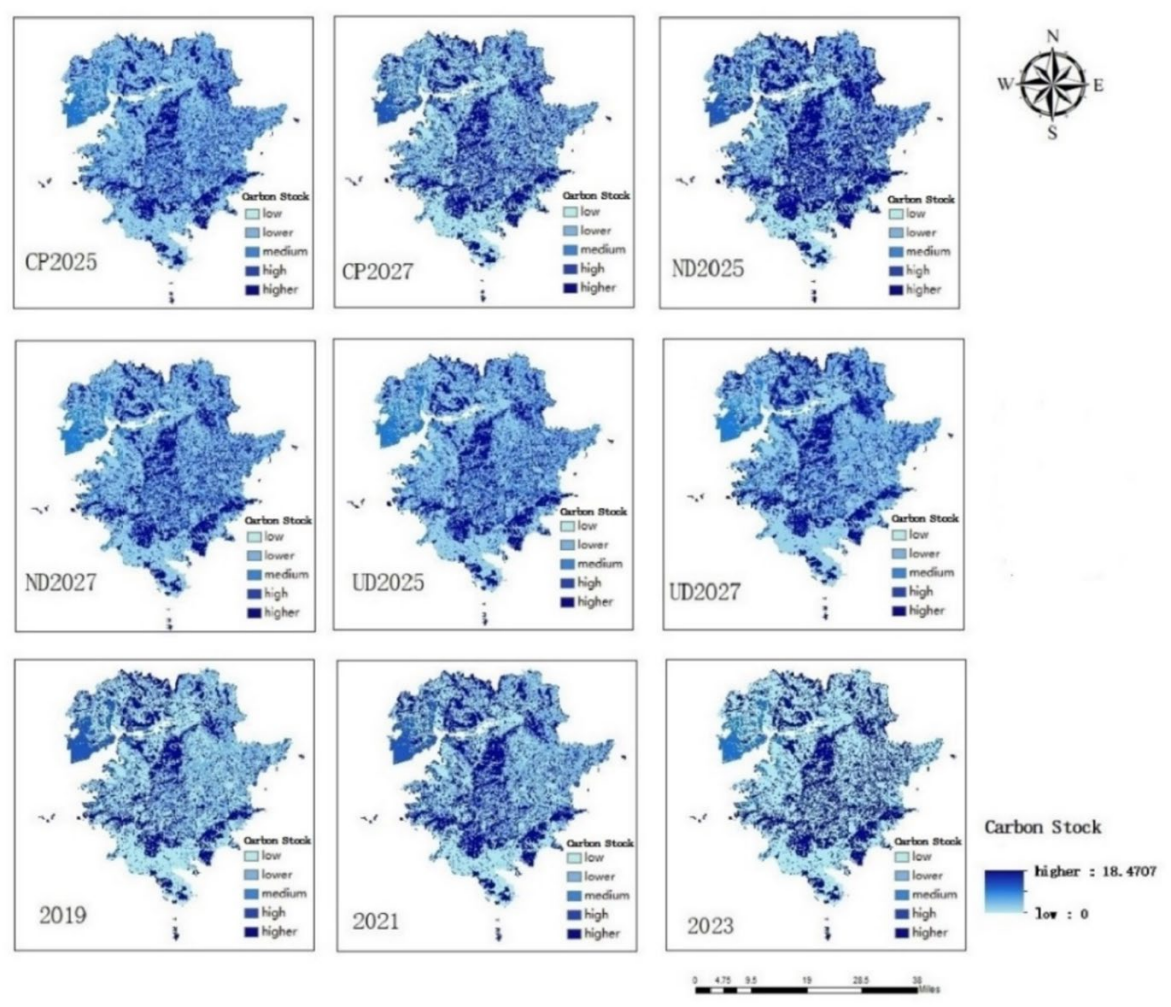
Spatial distribution of carbon stock in different years. (Figures 6 and 7 shows changes in carbon stocks under different development scenarios for 2019, 2021, 2023 and 2025 and 2027 obtained from forecast simulations and analyzed using the INVEST model, (1) an overall expansion of high-density regions of carbon stocks from 2019–2023. (2) Under different development scenarios, the higher carbon stock areas generally decrease in natural development and cropland protection scenarios and expand in urban development scenario).

**Fig. 7:**
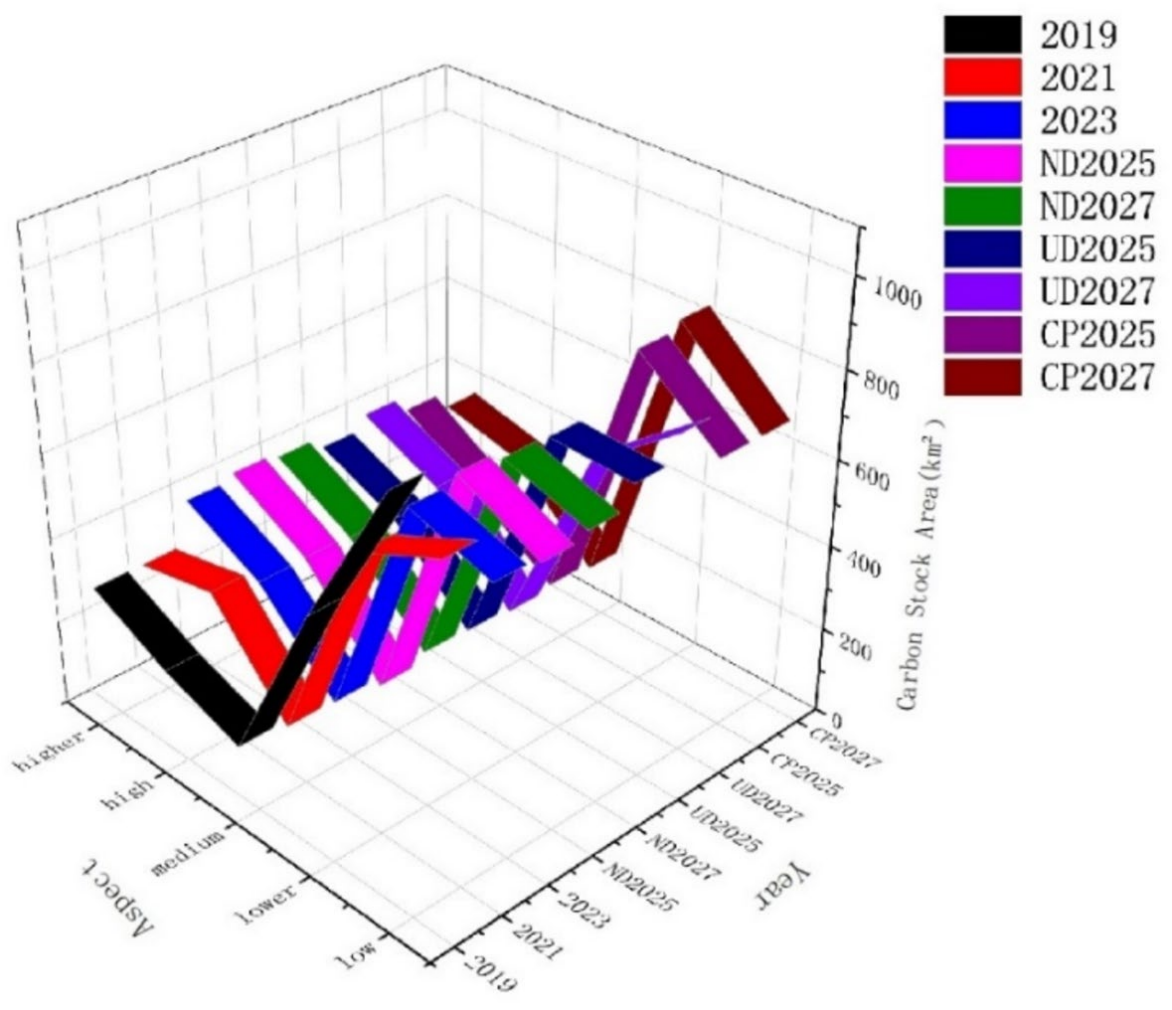
Changes of carbon stock area for different years.

Figure 1, 2, 4, 6, 8 Plotted by ArcGis10.8 (https://www.esri.com/), Figs. 3, 5, 7 Plotted by Origin2024 (https://www.originlab.com/).

## Research methodology

### Random forest algorithm with GEE support

The basic principle of Random Forest algorithm is an algorithm that integrates multiple trees through the idea of integrated learning, its basic unit is the decision tree, each decision tree is a classifier, then N trees will have N classification results. Random forest integrates all the categorization voting results and designates the category with the most votes as the final output, it can be easily trained in parallel.

Basis of research needs, the land use of 3 years, 2019, 2021, and 2023, is deciphered, and the Sentinel-2 images from April to November are chosen, and the B2, B3, B4, B5, B6, B7, B8, B8A, B11 bands of the Sentinel-2 images are selected to assist the NDVI, NDWI, and BSI, slope, elevation, etc. as other classification feature sets, sample point selection based on Google Earth Engine Sentinel satellite image maps, of which 250 sample points of water, the remainder of the other land class sample points were picked 500 (80% as a training sample, 20% as a validation sample), The number of samples obtained from the three-phase data is the same, paired with the chosen validation samples of the categorized. The number of samples taken in the three periods of data is the same, and the accuracy verification of the classification results is carried out with the selected validation samples. The overall classification accuracies of the classification results for the three periods of 2019, 2021, and 2023 are 90.31%, 90.42%, and 90.10%, and the accuracies of the Kappa coefficients are 86.3%, 87.8%, and 87.1%, which satisfy the requirements of the data accuracy.

### Carbon stock calculations supported by invest modeling

The Invest model, also called the Integrated Valuation of Ecosystem Services and Trade-offs (IVEST), gives decision makers a scientific basis for simulating changes in the number and value of ecosystem services beneath various land cover scenarios^4^ and can help assess the advantages and drawbacks of human activities^6^, scientific basis^6^, in which the carbon stock calculation module in the model. Ecosystem carbon stocks are separated into four primary carbon pools by the Invest model’s carbon stock module: above-ground biogenic carbon (carbon in all surviving plant material above the soil), below-ground biogenic carbon (carbon found in the root systems of live plants), soil carbon (organic carbon distributed in organic and mineralized soils), dead organic carbon (carbon in apoplastic material, inverted or standing dead trees)^9^, see supplementary documentation for specific values, viz:

1 $$C_{total} = C_{above + } C_{below + } C_{soil + } C_{dead}$$

In the above Eq. (1): ${C}_{total}$ denotes the overall carbon stock; ${C}_{above}$ denotes the above-ground carbon stock of vegetation; ${C}_{below}$ denotes the belowground carbon stock of vegetation; ${C}_{soil}$ denotes the soil carbon stock, ${C}_{dead}$ denotes the dead organic matter carbon stock.

The data of the carbon stock module in this paper comes from other scholars’ inquiry^25^, used to provide additional information, and the carbon stock density map of Jinpu New District is calculated.

### Landscape ecological risk index (ERI) construction and calculation

#### Landscape ecological risk index construction

The landscape ecological risk index includes degree of landscape disturbance, landscape vulnerability, landscape loss, landscape dominance and landscape separation^29,30^. The degree of landscape disturbance is used to reveal the degree of outside disturbance to the ecosystems in different landscapes^31^, and according to the landscape pattern analysis, the more disturbed area, where has the higher ecological risk, to construct the landscape disturbance index-${E}_{i}$, degree of disturbance of ecosystems in different landscapes through the superposition of indices^32^.

Landscape fragility-${F}_{i}$ denotes the vulnerability of the internal structure of ecosystems in different landscapes, and can reflect the size of the resistance of different landscapes to outside disturbances^33^. The less resistant a landscape type to external disturbance, the greater the vulnerability and it has the higher ecological risk. The differences in the resistance of distinct landscape types to external interference are correlated to the step of the natural succession process, the ecosystems primary succession stage have less resistance to external interference and are more fragile, and their indexes are constructed by using the expert scoring method and grading according to the realistic situation of the study area^32^.

The landscape loss index-${C}_{i}$ is constructed by superimposing different indices to reveal the degree of loss of natural attributes of ecosystems in different landscapes when they are subjected to both natural and anthropogenic disturbances^32^. The landscape dominance index is ${D}_{i}$, is the relative importance or degree of dominance of landscape elements within a given region, and the landscape separation index is ${N}_{i}$, indicates the degree of fragmentation of patches, reflecting the impact of external disturbances on the urban ecological networking, as shown in Table 1.

**Table 1:** Landscape ecological index construction formula.

Index	Formulas	Hidden meaning
Landscape separation index	${N}_{i}= \sqrt{\frac{{n}_{i}}{{2p}_{i}}}$	Where ${n}_{i}$ is the quantities of patches to ratio in landscape type i to the total area of patches in all land types,${p}_{i}$ is the area of patches to ratio in landscape type i to the total area of patches in all land types
Landscape dominance index	*D*_*i*_ = *L*_*i*_ * 0.6 + *p*_*i*_ * 0.4	Where ${L}_{i}$ is the quantities of patches to ratio in landscape type i to the total quantities of patches in all land types, with weights obtained from Ref
Landscape disturbance index	*E*_*i*_ = *H*_*i*_ * 0.5 + *N*_*i*_ * 0.3 + *D*_*i*_ * 0.2	Where ${H}_{i}$ is the patch density refers to the quantities of patches to ratio in landscape type i to the total area of patches, where the weights are obtained from the references and need to be summed up to one
Landscape fragility index	*F*_*i*_	The landscape fragility index is a classification of the ecological influence of each category, according to the geographic location of Jinpu New District, Dalian City, the construction land, arable land, forest land, grassland, and watersheds are classified as 1, 5, 3, 4, 2 in this order
Landscape loss degree index	*C*_*i*_ = *E*_*i*_ * *Y*_*i*_	Where ${E}_{i}$ is the landscape Disturbance Index. ${Y}_{i}$ is the normalized landscape fragility, which is the ratio of the result of subtraction between the landscape fragility index and the minimum value of landscape fragility in category i to the result of subtraction between the maximum of landscape fragility and the minimum of landscape fragility

Table 1 shows the formulas for the indices in the Landscape Ecological Risk Index (ERI) and the specific representation of each function in the formulas).

#### Calculation of landscape ecological risk index

Landscape ecological risk is an essential measure of how natural or manmade disturbances interact with the ecological environment and landscape pattern, expressing the link between man and land^14,21,34^, which is calculated by the formula:

2 $$ERI_{K} = \mathop \sum \limits_{i = 1}^{n} \frac{{A_{ki} }}{{A_{k} }} \times C_{i}$$

In the above Eq. (2): ${ERI}_{K}$ denotes the landscape ecological risk index, ${A}_{ki}$ denotes the landscape area of category i in the evaluation unit-k,${A}_{k}$ denotes the total landscape area in the evaluation unit-k, *n* denotes the all quantities of evaluation units, ${C}_{i}$ denotes the degree of landscape loss.

### PLUS modeling with multiple scenario settings

The PLUS model is a land use prediction model that can reliably simulate changes in different years and land use patches in diverse surroundings, and find the driving reasons^35,36^. In this paper, we simulate future land use changes under natural development scenario, urban priority development scenario, and cropland protection scenario in 2025 and 2027 using three phases of land use data. We select the GDP, population density, slope, DEM, and road network data of Jinpu New Area as influencing factors, and the water domain as limiting factors for prediction.

The research of this paper takes the national 14th and 15th Five-Year Plan as the time period, and Dalian Land Use Master Plan (2006–2020) and Dalian Territorial Spatial Master Plan (2021–2035) as the guiding outline, which mentions vigorously expanding the urban agglomerations and improving economic growth; it also mentions the problems of the current urban development such as less arable land resources and the prominent contradiction between people and land, and takes the protection of arable land resources as one of the important issues in the development process. Therefore, this paper chooses three development scenarios: Natural development, Urban development and Cultivated land protection scenario.

Scenarios-setting: Natural development scenario (ND): natural development scenario is that do not set conversion probabilities between various types of sites and do not take into account development policy requirements; Urban development scenario (UD): urbanization is a goal that China has consistently pursued, policy priority for urbanization in this scenario, and as the rate of urbanization accelerates in the future, people’s yearning for a better living grows. Considering the accelerated urbanization in the future, people’s demand for a better life is increasing; thus, The urban development scenario is designed to raise the probability by 20% of converting grassland, forest, and arable land to construction land, and to reduce the probability by 30% of shifting land for construction to other land types than arable land^37^; Cultivated land protection scenario (CP): Cultivated land safety is the foundation of ensuring food safety, This scenario prioritizes the development of policies to protect arable land, and the scarcity of farmland in the Dalian city area is more apparent, as is the conflict between people and land; thus, the cultivated land protection scenario is set to control cultivated land safety. This scenario decreases the transfer probability of restricting the converting of arable land to building land by 60% and the transfer probability to grassland by 40%, so tightly implementing the arable land conservation policy^38^.

Transfer probability formula:

3 $$\left({P}_{i-1}+ {P}_{i-2 }+ {P}_{i-3}+{ P}_{i-4} \right)*\text{X}+\left(1+D\right)=1$$

4 $$\left({P}_{i-1}+ {P}_{i-2 }+ {P}_{i-3}+{ P}_{i-4} \right)*\text{X}+1-\text{D})=1$$

In the above Eq. (3):${{\varvec{P}}}_{{\varvec{i}}-1} , {{\varvec{P}}}_{{\varvec{i}}-2 }, {{\varvec{P}}}_{{\varvec{i}}-3} ,{ {\varvec{P}}}_{{\varvec{i}}-4}$ are probability of shifting the land category i to the land categories other than those that need to be adjusted,* D* is the percentage of increase in the probability of transferring a certain land type to the other land types,and* D* is the percentage of decrease in the probability of shifting a particular land category to the other land categories in Eq. (4).

### Bivariate autocorrelation analysis of spatial conflicts

Spatial autocorrelation analysis is appropriate for measuring whether the distribution of spatial variables is agglomerative or not, and it can better define the link between geographic objects and assess the degree of aggregation or disaggregation among the properties of spatial elements of things^20^. Carbon stocks and landscape ecological risk distributions show typical spatial divergence characteristics as a result of land use change, and thus can be analyzed for spatial conflict aggregation by basic bivariate spatial autocorrelation methods, calculating the Moran Index, reflecting the spatial correlation between the variables, Moran’s index calculation formula is as follows:

5 $$I_{hk} = n\mathop \sum \limits_{i = 1}^{n} \mathop \sum \limits_{j = 1}^{n} W_{ij } *Y/\left( {n - 1} \right) \mathop \sum \limits_{i = 1}^{n} \mathop \sum \limits_{j = 1}^{n} W_{ij} \quad Y = \left( {\frac{{x_{{ih - \overline{x}_{h} }} }}{{Z_{h} }}} \right)\left( {\frac{{x_{{ik - \overline{x}_{k} }} }}{{Z_{k} }}} \right)$$

In the above Eq. (5): *I* is the Moran index whose value is between [− 1, 1], with 0 as the cut-off point, if the value is > 0, it indicates that the two present spatial positive correlation, and if the value is < 0, it indicates that the two are inverse correlation^39^. ${X}_{ih},{X}_{ik}$ are the values of the carbon stock density and the landscape ecological risk index of the evaluation unit, respectively. ${Z}_{h},{Z}_{k}$ are the variances of the carbon stock density and landscape ecological risk index, ${W}_{ij}$ is the weight matrix based on the two.

### Land use matrix and simulation accuracy

Transfer cost matrix: Based on the probability of each land class transferring like other land classes summing up to 1, use the transfer probability formula to recalculate the transfer probability of each land class, set up the land transfer cost matrix (Table 2) as well as the weighting ratio of each land class factor for simulation prediction, and calculate the carbon stock and ecological risk of landscape after obtaining land simulation results.

**Table 2:** Land use transfer cost matrix.

Scenarios	Natural					City					Cropland protect				
	a	b	c	d	e	a	b	c	d	e	a	b	c	d	e
a	1	1	1	1	1	1	0	0	0	1	1	0	0	0	0
b	1	1	1	1	1	1	1	1	0	1	1	1	1	0	1
c	1	1	1	1	1	1	1	1	1	1	1	1	1	1	1
d	0	0	0	1	0	0	0	0	1	1	1	0	0	1	0
e	0	0	0	0	1	0	0	0	0	1	0	0	0	0	1

Table 2 represents the land-use transfer matrix, showing the matrices for the conversion of each specific land category to each of the categories under different development scenarios, a represents city, b represents water, c represents tree, c represents grass, e represents farmland.)

Validation of simulation accuracy: In the paper, the study uses land use data from 2019 and 2021 to conduct the forecast simulation in 2023 under the natural development scenario, urban development scenario, and cultivated land protection scenario, respectively, and compares it to the actual 2023 land use data owned, and so on, and the accuracy of the model validation is 0.812, 0.801, 0.819, which is indicates that the simulation has a high degree of accuracy^35^.

## Results and analysis of results

### Land use results and multi-scenario simulation

According to the data source of this paper, the GEE cloud platform was used to process the land use data of the study area in 2019, 2021, 2023 to get the current land use status in 3 years, and use the PLUS model to simulate the future land use changes in 2025 and 2027; get the simulation results as shown in Fig. 2. The specific transformation of the area of each category is shown in the Fig. 3.

### Changing in landscape ecological risk and simulating results

We classified the landscape ecological risk (ERI) (Figs. 4 and 5, adopted the natural breakpoint method^32^ into five classes of low risk (ERI <= 0.243), lower risk (0.243 < ERI <= 0.404), medium risk (0.404 < ERI <= 0.550), high risk (0.550 < ERI <= 0.643), and higher risk (> = 0.643). According to the statistics, the higher-risk zone expanded substantially between 2019 and 2021, more pronounced in the central and eastern regions, and the percentage of high-risk and higher-risk zones increased by 0.98% and 2.31%, respectively. Between 2021 and 2023, the share of higher-risk zones decreased by 0.68%, while the share of high-risk zones increased by 0.43%. According to the change in the percentage of risk areas, from 2019 to 2021, the percentage of higher-risk and high-risk areas increased, and the land use changes clearly show a correlation, and the increase and decrease between 2021 and 2023 are successively reduced, and some areas of higher-risk areas in 2023 have significantly decreased, and the increase in the percentage of high-risk areas is obvious, which indicates that higher-risk areas under this land planning and policy still showing an increasing trend.

In 2025, the natural development scenario indicates a decrease in the high risk areas compared to 2023. The high risk areas decrease by 4.02% to 91.64 km^2^, while the higher risk areas increases by 0.43% to about 9.73 km^2^, and decrease in the high risk areas by 0.72% in 2027 compared to 2025, to about 16.21 km^2^ and decrease of 0.97% to about 22.02 km^2^ in the higher risk areas, from the common risk projections in 2025 and 2027. For natural development scenarios, the overall trend is still decreasing compared to 2023, but the trend of the higher risk trend to become lower is smaller and the trend of the low risk area to become larger is improved in 2027 based on 2025, which may be related to the fact that some of the 2027 in the natural development scenario land such as the uplift in built-up land area is effectively utilized. (2) The urban development scenario reduces the high risk areas by 2.74% to about 62.47 km^2^, and the higher risk areas by 0.59% to about 13.52 km^2^ in 2025 compared to 2023. The higher risk areas are significantly reduction in 2027, mainly in the central region, high risk areas has an increase of 2.47% to about 56.43 km^2^ in 2025, and an increase in the higher risk area of 1.73% to about 39.34 km^2^. For the urban development scenario, the overall high-risk areas in 2025 is conspicuously reduced and the low-risk areas is increased, the higher-risk areas in 2027 is decreased from 2025, it may be relevant to the large-scale development of the city and ecology, that is related to the effective use of part of the cultivated land as well as the unutilized land in the cultivated land. (3) Cultivated land protection development scenario increases by 1.86% in 2025 compared to 2023 compared to the high risk areas, which is about 42.47 km^2^, and the higher risk areas increase by 1.24% compared to 2023, which is about 28.39 km^2^, and the high risk areas decrease by 2.28% in 2027 compared to 2025, which is about 52.08 km^2^, and the higher risk areas increase by 2.15%, which is about 49.04 km^2^. The trend of higher risk areas in 2025 and 2027 presents a trend of increasing, and the decrease in high risk areas, and the increase in 2027 may be relevant to the continuous expansion of cropland and neglecting the ecological protection of the land resulting in a higher risk.

### Changing in carbon stocks and simulating results

According to the data situation (unit t/km^2^), Carbon stock density is classified into five categories (low, lower, medium, high, and higher) based on data (Figs. 6 and 7) show that the higher carbon stock areas are mainly in central, southern, part of southwestern and northern parts of the region, which have better ecological protection and widely distributed forests and grassland. And that the high-density areas increase by 5% between 2019 and 2021, which is about 127.86 km^2^. The decrease in the higher-density area is smaller, about 0.49 km^2^, and the overall carbon sink becomes higher, reflecting the overall better protection of the ecological environment in this time period; between 2021 and 2023, the high-density areas decrease by 1.45%, about 33.12 km^2^, and the higher-density areas increase by 4.39%, about 100.10 km^2^, and the carbon sink increases, with an overall rise in ecological quality.

Natural development scenario: in 2025 compared with 2023, the high density areas increase by 0.13%, about 2.97 km^2^, and the higher density areas increase by 0.57%, about 12.99 km^2^, and in 2027 compared with 2025, the high density areas decrease by 0.24%, about 5.46 km^2^, and the higher density areas decrease by 0.52%, about 11.93 km^2^. The natural development scenario The decrease in 2027 from 2025 under the natural development scenario has an even distribution of areas and an overall decrease in higher carbon areas, the main reason for which may be related to the Growth of construction land in 2027 under this development scenario, and the successive decrease in other land types except for waters. (2) City development scenario: in 2025 compared to 2023, the high density areas decrease by 0.55%, about 12.52 km^2^, and the higher density areas decrease by1.14%, about 25.96 km^2^, and in 2027 compared to 2025, the high density areas increase by 1.11%, about 25.29 km^2^, and the higher density areas increase by1.35%, about 30.74 km^2^. The town development scenario shows that the 2025 to 2027 the overall higher carbon area are increasing, concentrated in the central mountainous area and the increase is obvious, may be in the process of urbanization occupied part of the ecological area, and then focus on the expansion of the construction of the central ecological reserve, the forest land area in 2027 than in 2025 increased significantly. (3) Cultivated land protection scenario: in 2025 compared with 2023, the high density areas decreased by 2.40%, about 54.65 km^2^, and the higher density areas decreased by 1.38%, about 31.42 km^2^. In 2027 compared with 2025, the high density areas increased by 1.43%, about 32.56 km^2^, and the higher density areas decreased by 1.74%, about 39.62 km^2^. Cultivated land protection and development scenarios of the higher carbon areas are overall reduction, may be the continuous expansion of cultivated land, breaking through the red line with the ecological reserve, so that part of the ecological reserve to cultivated land, which leads to the reduction of higher-carbon areas.

### Spatial conflict distribution results

**Fig. 8:**
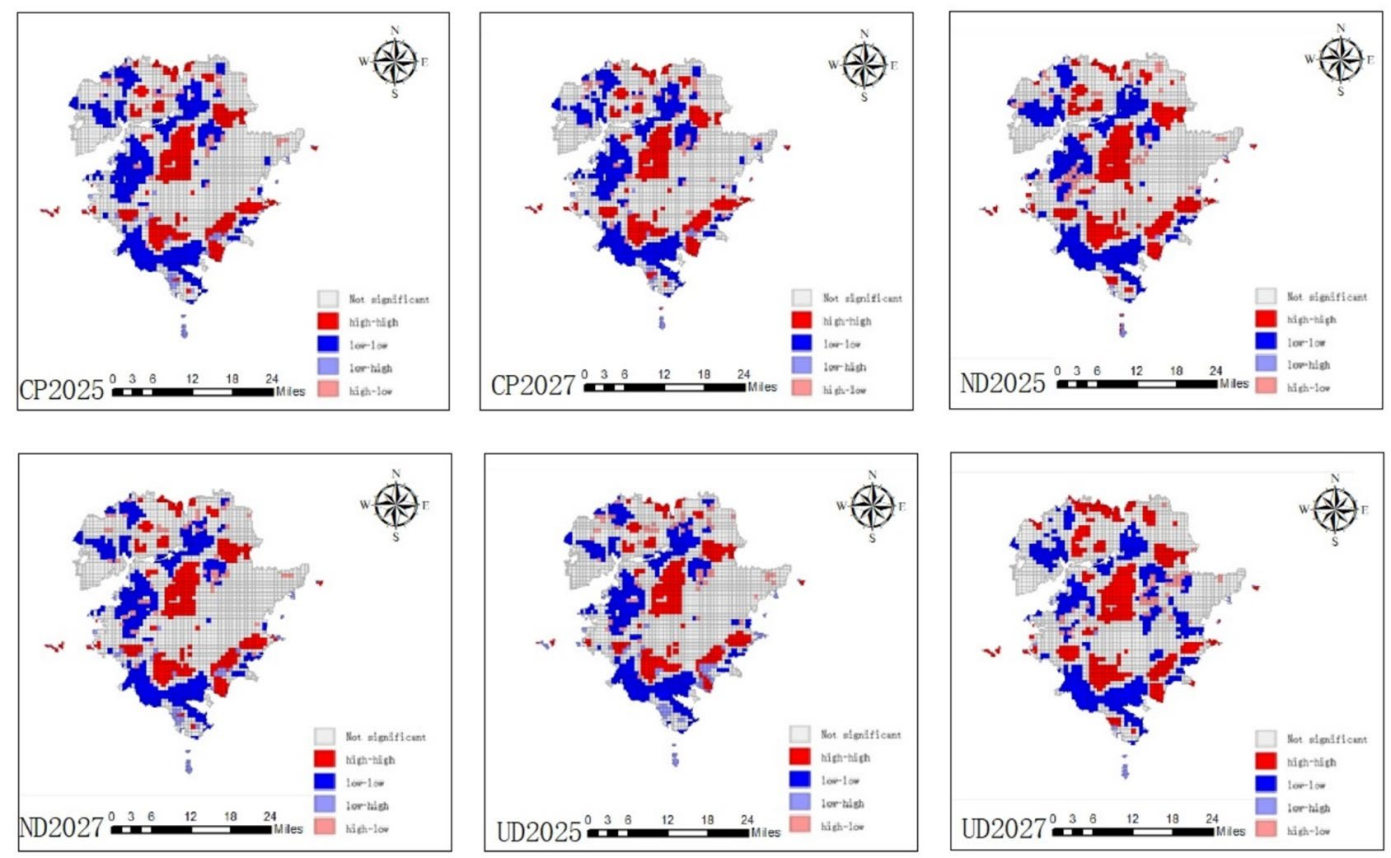
Spatial distribution of conflict results under multiple scenarios. (Figure 8 shows the spatial autocorrelation results obtained from the prediction simulation, using the local spatial autocorrelation analysis in GeoDa software, and the calculation results show that the conflict between carbon stocks and high values of ecological risk in the landscape is obvious and mainly distributed in the central and southern regions, and the conflict between the three scenarios is obvious in the northern region, Differences in UD scenario conflicts are more pronounced).

The spatial autocorrelation analysis of the spatial and temporal variations of the predicted changes in carbon stock and landscape ecological risk in Jinpu New Area in 2025 and 2027 (Fig. 8) was carried out to analyze the differences in the distribution of the spatial conflict relationship between the two under different development scenarios, with the values of Moran’s I for the natural development scenario being 0.468 and 0.317, for the urban development scenario 0.364 and 0.481, and for the arable land protection development scenario 0.375 and 0.384. 0.364, 0.481, and the values of Moran’s I under the scenario of cultivated land protection and development are 0.375, 0.384, which can be seen as a positive correlation between the conflict relationship, which indicates that the distribution of the two in the space is not completely random, in the spatial correlation, the high-high indicate that the region with high and higher carbon stock and the landscape ecological high and higher risk are adjacent to each other, the low-low indicate that the region of low and lower carbon stock and the region of low and lower risk are adjacent to each other, and the hotspot of the conflict area for high carbon—high risk Clustering, according to the results of the map comparison shows that the three different development scenarios of conflict hotspots in the same area are centered on the central, south-central, eastern and northeastern part of the region, the conflict relationship between the differences in the region appeared in the northern and western part of the region, which the town scenario 2027 compared to 2025 compared to the east of the cold spot area significantly reduced, and with the continuous urbanization, and the expansion of urbanization related to conflict hotspot areas than the 2025 there is a significant increase in the number of conflict hotspot areas.

## Conclusion

This study investigates land use changes in Jinpu New District of Dalian City in recent years, and simulates land use projections of Jinpu New District in 2025 and 2027 under three scenario (natural development, urban priority development, cropland protection), evaluates carbon stock changes in present years by using the InVEST model, to forecast carbon stock changes and the landscape ecological risk changes under the different scenarios of the development mode by the PLUS model and INVEST model. Ultimately, use the spatial autocorrelation analysis to determine the distribution of spatial conflict regions between the two. Conclusions are taken from this study:

(1) Land use change’s effects on carbon stocks and the landscape ecological risk in Jinpu New Area: (1) The overall increase of higher carbon stock and high density area from 2019 to 2023 is about and 4.37% and 4.16%, the proportion of both higher risk area and high risk area between 2019 and 2023 is overall increase, respectively 4.71% and 1.77%, the dominant land type is the increase of cultivated land, forest land and the decrease of construction land, the decrease of construction land during the period of construction land, part of the land that is not well During the decrease of construction land, part of the land is not well utilized or reasonably cultivated, which leads to the evolution of the areas that should be reasonably planned to weeds, wasteland and unused land, and increases the ecological risk of some areas to become higher. (2) From the model of the different future development scenarios in 2025 and 2027, Under the natural development scenario, the natural development scenario shows that carbon stocks in the higher density zone and the high density zone increase and then decrease, with an overall decrease of very little magnitude, while the high risk zone is decreasing, at about 4.73 per cent, and the higher risk zone increases and then decreases, with an overall decrease of about 0.54 per cent; under the urban development scenario, the carbon stock is predicted to a total increase of 1.12% and 1.35% km^2^ in the high and higher density zones, and the landscape ecological higher-risk and high-risk zones is significantly reduced in 2025 compared to 2023, while the higher risk zones in 2027 compared to 2025 show an increasing trend instead, which is about 5.78%; under the cropland protection scenario, from 2023 to 2025 and 2027, the predicted density of carbon stock shows a decrease in higher-density area, and the landscape ecological higher-risk area in 2025 compared to 2023 decreases significantly, while the higher-risk area in 2027 under this development mode instead increases compared to 2025. (2) Conflict relationship analysis and management in Jinpu New Area: The results of autocorrelation analysis indicate that the significant areas of conflict relationship under the three different development scenarios are the central, south-central, eastern and part of the northeastern part of the area, and the hotspots in the north and part of the east where the difference in the conflict relationship is obvious, in which the conflict distribution area of the urban priority development scenario is obviously more than that of the natural development and cultivated land protection development scenario, so the urban priority development scenario is not suitable for the future land use development mode in the study area.

## Discussion

1. Land use planning and management play a crucial role in promoting carbon peak and carbon neutrality goals^6,25,40^. Many scholars have confirmed the accuracy of the PLUS model in simulating land use change and the INVEST model in calculating carbon stock^41,42^, and several studies have shown that there is a link between land use change and the two indicators of carbon stock and landscape ecological risk, e.g., Yuan Xue^35^ et al. used the PLUS model to simulate the scenario of land use change in Wuhan and study the ecological risk of the landscape, and Li Jinpu^6^. Research on land use change and ecosystem carbon stock using InVEST model, Xia Shengjie^39^ et al. Spatial autocorrelation analysis of dynamic evolution of ecological land use and thermal environment in urban agglomerations using ArcGIS-GeoDa.This research paper combines GEE, PLUS, Invest, and Geoda modules to develop a conflict-coupled model of carbon stocks and landscape ecological risk as influenced by land use change., and analyses the distribution of spatial conflict relationships in response to effects of changing land use on carbon stock and landscape ecological risk in some coastal cities with little arable land and land use constraints. From the 3 years of land use changes in 2019, 2021, 2023, the paper investigates the conflict relationship between carbon stock and landscape ecological risk in the ecological space, and provides a reference basis for how to rationally plan the spatial resource pattern of the land and promulgate the relevant land policies in the process of urbanization^43,44^, to maintain the capacity of carbon sinks while also reducing ecological risk, and to narrow down the hotspots of conflict. Combined with the results of the data in this paper, based on the features of various development situations, the future carbon stock and ecological risk show different variations, Among them, the urban development scenario has the widest distribution of conflicts than the other two while the natural development and cropland protection scenarios have fewer conflicts overall, but the natural development scenario has uncertainty, and the cropland protection scenario can maintain a certain amount of carbon stock but the ecological risk tends to become higher, so it is possible to optimize the land use policy and management by combining the respective characteristics of the natural development scenario and the cropland protection scenario.
2. Policy recommendations :The results of this paper show that when the central, southern and western regions of the mountains and forests to establish ecological reserves, to prevent the degradation of forest to grassland and farmland, at the same time the speed of urbanization in the future, in the development of urbanization at the same time, but also to guard against carbon loss, rational planning of the land, protection of arable land, demarcation of ecological protection zones and arable land and built-up land of the red line, to guard against the degree of man-made ecological destruction of urbanization; to scientifically formula the boundaries of urbanization. For instance, in this paper ERI scenario setting, grassland areas with high risk, it has a low carbon stock, can be appropriate to prevent the ecological damage. The risk of grassland area is high, the carbon stock is low, and it can be transformed between arable land and forest land appropriately, to improve the overall degree of landscape while guarding against carbon loss, to mitigate the tendency of high-value agglomeration in some areas, to control the land for construction, to protect the arable land and to demarcate the red line with the ecological protection zone of the forest, and to efficiently utilize the land. The changes under different development scenarios have different trends, but for any development scenario, ecological restoration and re-planning of concentrated part of the conflict hotspot areas to reduce the trend of conflict intensification and improve the quality of urban ecology is still the main,.
3. Research limitations and future perspectives: The research in this paper also has certain limitations, such as the use of supervised classification methods to obtain land use data may exist in the accuracy and manual selection of the sample error and other aspects of the problem, resulting in data accuracy is not high enough, while the use of ecological indicators also has certain limitations, this paper only for the relationship between carbon stocks and ecological risk on how to promote the hot spot of the conflict to maintain the capacity of carbon sinks, reduce the ecological risk of the region, to the high-carbon -This paper only provides a reference for the relationship between carbon stock and ecological risk on how to promote the conflict hotspot areas to maintain carbon sink capacity, reduce regional ecological risk, and transform to high carbon and low risk.

## Supplementary Information

Supplementary Information.

## Data Availability

The datasets generated during and/or analysed during the current study are available from the corresponding author on reasonable request.
